# Illumination discrimination in real and simulated scenes

**DOI:** 10.1167/16.11.2

**Published:** 2016-09-01

**Authors:** Ana Radonjić, Bradley Pearce, Stacey Aston, Avery Krieger, Hilary Dubin, Nicolas P. Cottaris, David H. Brainard, Anya C. Hurlbert

**Affiliations:** radonjic@sas.upenn.edu http://www.sas.upenn.edu/~radonjic/; bradmpearce@gmail.com; stacey.aston1@newcastle.ac.uk https://staceyaston.com/; avekrieger@gmail.com; hdubin@sas.upenn.edu; cottaris@sas.upenn.edu https://color.psych.upenn.edu/people/nicolas-p-cottaris; brainard@psych.upenn.edu https://color.psych.upenn.edu/people/brainard; anya.hurlbert@newcastle.ac.uk http://www.ncl.ac.uk/ion/staff/profile/anyahurlbert.html; Department of Psychology, University of Pennsylvania, Philadelphia, PA, USA; Institute of Neuroscience, Newcastle University, Newcastle upon Tyne, UK

**Keywords:** *color vision*, *color constancy*, *illumination perception*, *illumination discrimination*, *computer graphics*, *real vs. simulated scenes*

## Abstract

Characterizing humans' ability to discriminate changes in illumination provides information about the visual system's representation of the distal stimulus. We have previously shown that humans are able to discriminate illumination changes and that sensitivity to such changes depends on their chromatic direction. Probing illumination discrimination further would be facilitated by the use of computer-graphics simulations, which would, in practice, enable a wider range of stimulus manipulations. There is no a priori guarantee, however, that results obtained with simulated scenes generalize to real illuminated scenes. To investigate this question, we measured illumination discrimination in real and simulated scenes that were well-matched in mean chromaticity and scene geometry. Illumination discrimination thresholds were essentially identical for the two stimulus types. As in our previous work, these thresholds varied with illumination change direction. We exploited the flexibility offered by the use of graphics simulations to investigate whether the differences across direction are preserved when the surfaces in the scene are varied. We show that varying the scene's surface ensemble in a manner that also changes mean scene chromaticity modulates the relative sensitivity to illumination changes along different chromatic directions. Thus, any characterization of sensitivity to changes in illumination must be defined relative to the set of surfaces in the scene.

## Introduction

Variations in illumination are pervasive in natural viewing. The light in the environment changes in color and brightness over the course of the day and with variations in atmospheric conditions (Judd, MacAdam, & Wyszecki, 1964). Similarly, the light changes across a scene as it interacts with objects, creating shadows and interreflections (Nascimento, Amano, & Foster, 2016). Such temporal and spatial changes in illumination introduce a challenge for the visual processing of object color, as the light reflected from objects to the eye depends not only on object surface reflectance (a physical correlate of color), but also on the spectral composition of the incident illumination. Despite changes in illumination (and, hence, in the reflected light), the visual system maintains a reasonably stable representation of object color, a phenomenon known as *color constancy*.

Efforts to understand color constancy typically focus on measuring the stability of surface color appearance across changes in illumination (Hurlbert, 1998b; Smithson, 2005; Foster, 2011; Brainard & Radonjić, 2014), while perception of spatial and temporal changes in illumination per se has been less studied (but see, for example Katz, 1935; Beck, 1961; Craven & Foster, 1992; Rutherford & Brainard, 2002).

Recently, Pearce, Crichton, Mackiewicz, Finlayson, and Hurlbert (2014) developed a novel paradigm for probing color constancy by assessing individuals' sensitivity to changes in illumination. The logic behind their operationalization is that an individual's inability to detect illumination changes in a scene that consists of a fixed set of surfaces likely indicates the stable color appearance of these surfaces. Under this interpretation, color constancy may be quantified by the extent of an illumination change that can occur without being discerned; thus, a high discrimination threshold for an illumination change would correspond to a high level of color constancy. In their study, Pearce et al. (2014) measured illumination discrimination ability along four different chromatic directions (relative to a neutral 6700°K daylight): blue and yellow, which roughly capture the illumination variation typical of natural viewing, and red and green, which are less typical and which were orthogonal to the blue–yellow axis. Illumination changes were parametrized in a nominally uniform color space. They found that sensitivity differed across chromatic directions and that thresholds were the highest for the blue illumination change direction. Pearce et al. (2014) interpret this result as evidence that color constancy operates more robustly across illumination changes that are typical of natural viewing.

Although the logic described above makes plausible that sensitivity to changes in illumination is related to the stable perception of object color, we note that such a connection is not guaranteed a priori: it is in principle possible that illumination discrimination depends on a representation of illumination that is processed separately from the representation of object color. Linking illumination discrimination to color constancy requires experiments in which illumination discrimination and object color perception are explicitly measured for a common set of stimuli. Such experiments remain an important direction for future research. Nonetheless, illumination discrimination is an important and understudied perceptual ability in its own right and a number of interesting questions about its fundamental properties remain open. For example, although similar patterns of thresholds across different chromatic directions were found for a small number of scenes with different backgrounds and contents (Pearce et al., 2014), in general, little is known about how illumination discrimination depends on various stimulus characteristics (e.g., the ensemble of surfaces in the scene). Finally, the mechanisms underlying illumination discrimination, as it has been measured, are currently not well understood. One possibility is that that observers' performance in the illumination discrimination task is based on a distinct perceptual representation of scene illumination. Alternatively, individual observers might be tracking changes in lower-level image features or in the color appearance of individual surfaces in the scene. Pursuing these questions using real illuminated scenes as stimuli is challenging. For example, testing whether observers complete a task by tracking the appearance of individual surfaces would require frequent changes in the scene surface ensemble during the course of the experiment (to prevent such tracking). Similarly, contrasting sensitivity to changes in illumination to sensitivity to (well-matched) changes in surface color would require manipulation of object surface reflectance in parallel with manipulation of illumination. Thus, it is attractive to use computer-graphics simulations of three-dimensional scenes as stimuli to increase the range of stimulus manipulations that can readily be implemented. Note, however, that there is no assurance that measures of illumination discrimination in experiments that use even high-fidelity simulated scenes will match those obtained with real scenes. To explore this, we directly compared observers' performance in the illumination discrimination task for two types of stimuli across two groups of observers (Experiment 1). When the simulated and real scene stimuli were roughly matched in mean chromaticity and scene geometry, the measured illumination discrimination thresholds were essentially the same and varied across different chromatic directions in both conditions in a manner similar to that found by Pearce et al. (2014).

Capitalizing on this result, we then used simulated scenes to ask whether differences in sensitivity to changes in illumination across different chromatic directions are preserved when the surfaces in the scene, and hence the mean scene chromaticity, are varied (Experiment 2). Our results show that variation in scene surface ensemble modulates the relative sensitivity to illumination changes along different directions.

## Experiment 1

The experimental design was similar to that introduced by Pearce et al. (2014), but employed an adaptive staircase method rather than the method of constant stimuli. On each trial, observers discriminated between a scene viewed under two different illuminations—a target and a comparison. Across trials, the difference between the comparison and the target illumination varied, and illumination discrimination thresholds were extracted from the data. Thresholds were measured for the same four chromatic directions as in the Pearce et al. (2014) study: blue, yellow, green, and red.

One can develop an intuition about the task by examining rendered scenes under different comparison illuminations (see Figure 3 below). When the difference between the target and comparison illumination is small (1 CIELUV Δ*E* unit), distinguishing the comparison and target illumination is difficult and performance is expected to be near chance. The difficulty can be appreciated by examination of the top row of the figure, where the four distinct comparison scenes appear identical to each other at 1 Δ*E* illumination difference, and would also appear identical to the target. When the difference is large (50 Δ*E* units), discrimination is easy: the four comparison scenes are easily distinguished from each other, and would also be easily distinguished from the target. Thresholds lie between the two extremes. The middle row shows comparison scenes for a difference of 15 Δ*E* units, which is at the high end of our measured thresholds.

In the real scene condition, observers viewed a scene consisting of a box whose walls were covered with a Mondrian-patterned paper and in which the illumination was controlled via tunable multichannel LED light modules. The experimental setup was similar to that used by Pearce et al. (2014), but it used different spectrally tunable multichannel LED light modules. In the simulated scene condition, observers viewed a well-matched graphics simulation of the real scene, presented stereoscopically on computer-controlled monitors. Different groups of observers participated in the two conditions. The two experimental conditions were conducted at different locations (real scene: Newcastle University, UK; simulated scene: University of Pennsylvania, USA).

### Methods

#### Experimental setup—real scene condition

##### Apparatus:

**Figure 1:**
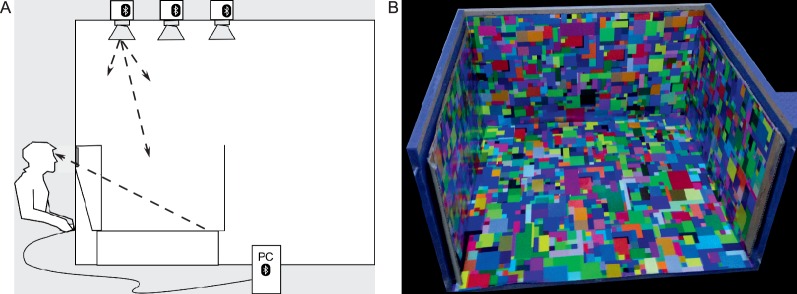
Apparatus in the real scene condition. (A) Diagram of the light room with the stimulus box (not to scale). The box was located flush to the front wall of the lightroom. Tunable LED light modules mounted in the ceiling illuminated the inside of the box. The observers viewed the box through a porthole in the front wall. (B) Uncalibrated photograph of the stimulus box lined with the Mondrian poster (the view shown here differs from observers' view in the experiment).

The stimulus box (47 cm high, 71 cm wide, 77 cm deep) was placed inside a custom-built lightroom (2.5 m × 2.5 m × 2.5 m; Figure 1A). The light room also housed the LED light modules, which illuminated the stimulus box. With its walls, ceiling, and floor painted in highly reflective white paint, the lightroom served as an integrating chamber. The neutral gray walls of the stimulus box were lined with a large-format inkjet-printed matte paper poster of a Mondrian pattern (Figure 1B). The Mondrian pattern consisted of a random distribution of rectangular surfaces that varied in size (0.2–12 cm on each side) and reflectance. The Mondrian pattern was constructed by digitally drawing overlapping rectangles into an image until it was fully covered, and assigning each rectangle a color sample at random from a set of distinct RGB values, chosen so that their chromaticity coordinates were above a minimum saturation level (i.e., perceptually nonneutral). Observers viewed the stimulus box through a small porthole (7.5 cm height × 14.5 cm width). Their field of view was restricted so that they were able to see only the inside of the stimulus box.

The stimulus box was illuminated by three identical spectrally tunable multichannel LED light modules (Omicron Lighting/Ledmotive LT-01 prototype luminaire; Ledmotive, Barcelona, Spain). The spectral power distribution of the resulting illumination emitted by the modules was controlled in real time by a Windows 7 computer (Intel i7 processor, 64-bit instruction set) via a Bluetooth connection (v2). Each light module contained 13 unique LED channels. Each channel was driven by an independent circuit and emitted light within a different wavelength band. The peak wavelengths of the set of channels covered the visible spectrum. The gamut of possible spectra emitted by the module could therefore be described as all nonnegative linear combinations of a set of 13 basis functions, each given by the spectral power distribution of light emitted by the corresponding LED channel. The spectra emitted by the light module were controlled by weights specifying the current delivered to each LED channel, and therefore specifying the spectral radiance of the channel's emitted light. The output of each channel was controlled with 16-bit precision. To approximate any desired illumination spectrum, we used quadratic programming to generate the set of appropriate weights for a well-fitting but spectrally smooth spectral match. The general procedures used to measure individual LED spectra and find the set of weights that generate a desired illumination are described in more detail in previous reports (Finlayson, Mackiewicz, Hurlbert, Pearce, & Crichton, 2014; Pearce et al., 2014). The experimental programs were written in MATLAB (MathWorks, Natick, MA).

##### Stimuli:

We generated 205 experimental illuminations using the procedure described above: the target illumination, which was a metamer of daylight of color temperature 6700K (D67) and 204 comparison illuminations (51 illuminations in each of the four chromatic directions of illumination change: blue, yellow, red, and green). All illuminations were roughly matched in illuminance but varied in chromaticity. The mean CIE xy chromaticity of the scene under the target illumination, estimated from a hyperspectral image, was [0.322, 0.349], and its mean luminance was 6.8 cd/m^2^. The hyperspectral image (96 planes, 400–780 nm at 4 nm resolution) was taken using a calibrated spectral camera (Specim V10E VNIR; Specim, Oulu, Finland); each pixel corresponded to 1.8 mm^2^ on the back wall of the box.

The chromaticities of the comparison illuminations in the blue and yellow illumination change directions were taken from the Planckian locus, which closely follows the daylight locus. The chromaticities of the comparison illuminations along the red and green directions were taken along a line orthogonal to the Planckian locus in the uniform chromaticity plane at 6700 K, computed according to the method established by Mori et al. (Wyszecki & Stiles, 1967); this is by definition the line of correlated color temperature to 6700 K. Along each chromatic direction, the u* v* chromaticities of comparison illuminations were chosen so that the amount of difference relative to the target illumination increased gradually from 0 to 50 CIELUV Δ*E* units, in steps of approximately 1 Δ*E* (see Figure 3). We estimate that, on average, 1 perceptual just-noticeable-difference (JND) corresponds to 4.5 CIELUV Δ*E* units. Our estimate uses the method described by Brainard (2003, p. 203), in which the size of the MacAdam ellipses was expressed in CIELAB Δ*E* units. Here we repeated the same calculation in CIELUV Δ*E* units. In this calculation, the relation between CIELUV Δ*E* and 1 JND is based on the assumption that 1 JND corresponds to 1.96 standard deviations of the ellipses. Although Wyszecki and Stiles (1982, p. 310) assert that a JND corresponds to 3 standard deviations of the ellipses, they note that 1 standard deviation has been the more commonly used value. Our choice of 1.96 is intermediate between these two plausible extrema.

In the real scene experiment we used only 50 of the comparison illuminations in each illumination direction (nominally 0 to 49 Δ*E*). The Δ*E* difference of each comparison illumination from its neighbors deviated from the nominal step of 1 Δ*E*. Mean absolute deviation from the nominal value, averaged across all illuminant steps and directions was 3.38 in both conditions (standard deviation was 1.99 for the real and 1.82 for the simulated condition). A table specifying the actual difference of each comparison illumination from the target in Δ*E* units is available in the online supplement, with separate tables provided for the real and the simulated scene conditions (http://color.psych.upenn.edu/supplements/illuminationdiscrimination1/). In the data analysis (see below) we used actual, rather than nominal, illumination differences.

##### Calibration:

A polymer white reflectance tile was placed at the back wall of the viewing box, with the Mondrian card removed and the neutral gray walls therefore uncovered. Each LED primary was displayed at maximum power in isolation, with spectra reflected from the tile measured from outside the lightroom through the porthole. Spectra were measured using a PR-650 SpectraScan radiometer (PhotoResearch, Chatsworth, CA) positioned so its lens was approximately at the observer's pupil plane and with it focused on the calibration tile. The average (across illumination spectra) luminance of a white calibration tile placed inside the viewing box was 24.4 cd/m^2^.

#### Experimental setup—simulated scene condition

##### Apparatus:

Stimuli were presented stereoscopically via a custom-made stereo-rig (for detailed description, see Lee & Brainard, 2014). The rig consisted of two calibrated LCD color monitors (24 in. NEC MultiSync PA241W) driven at a pixel resolution of 1920 × 1200, a refresh rate of 60 Hz, and with 8-bit resolution for each RGB channel via a dual-port video card (NVIDIA GeForce GT120). The observers viewed the displays through two rectangular apertures (2.7 × 2.5 cm) in a single black metal plate. The position of the apertures relative to the screens was such that the left screen was visible only to the left eye while the right screen was visible only to the right eye. The optical distance of each monitor to the eye was 76.4 cm. The host computer was an Apple Macintosh with an Intel Xeon quad-core processor. The experimental programs were written in MATLAB (MathWorks, Natick, MA), using routines from Psychtoolbox (Brainard, 1997; Pelli, 1997; http://psychtoolbox.org) and mgl (http://justingardner.net/doku.php/mgl/overview).

##### Stimuli:

**Figure 2:**
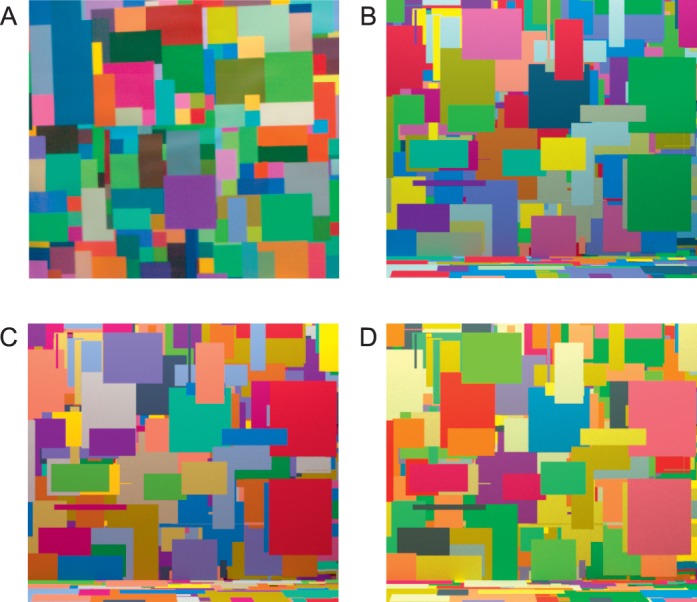
Rendered hyperspectral images showing the stimulus scene under the target illumination. (A) The real scene condition (Experiment 1). The image shown is cropped to show only a portion of a back wall of the stimulus box and does not correspond to observers' full field of view. (B) Simulated scene condition from Experiment 1 (equivalent to the neutral scene in Experiment 2). (C, D) The reddish-blue scene (C) and the yellowish-green scene (D) used in Experiment 2. Images are tone-mapped for illustration purposes as described in methods.

The stimuli were graphics renderings of a scene that was similar to the real scene (Figure 2A). A three-dimensional model of the stimulus box was designed in Blender (https://www.blender.org/). The back wall and floor of this box were covered with a Mondrian-like pattern of rectangular surfaces. This pattern was produced by randomly placing 2,000 overlapping rectangles, whose dimensions were also chosen randomly, until the visible portion of the back wall and the floor were fully covered with surfaces.

**Figure 3:**
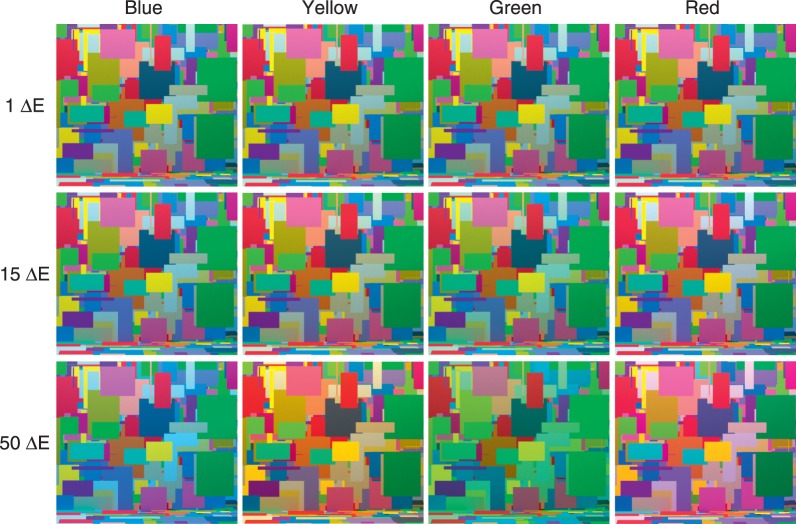
Simulated scene under different comparison illuminations. For each of the four illumination directions, we show the stimulus image (the left eye image from each stereo pair) rendered under comparison illuminations, which differ from the target by 1, 15, or 50 (nominal) Δ*E* units.

Each individual surface on the back wall and floor was assigned a reflectance function that corresponded to one of 16 preselected surface reflectance functions. This sample set of 16 was chosen from the many surfaces used in the stimulus box in the real scene condition, so that each of the surfaces were perceptually easy to distinguish from one another.

The stimulus box was illuminated by four square rectangular area lights positioned parallel to the ceiling, outside of field of view of the cameras used for rendering. A Blender file specifying the geometry of the stimulus scene is available in the online supplement.

The illumination spectra used for rendering corresponded to those measured in an earlier experiment (Pearce et al., 2014), which employed Gamma Scientific RS-5B multichannel LED modules (Gamma Scientific, San Diego, CA). The measurement procedures corresponded to those described in the earlier paper. These illumination spectra were essentially metamers of those produced with the luminaires used in the real scene condition of this experiment (they were generated to match the same set of chromaticities but with different LED spectral basis functions).

To create a simulated scene that matched the real scene in chromaticity, we first created 200 sample scenes by randomly assigning surface reflectance samples from the set of 16 to the surfaces in the scene and then rendering them under the target illumination. For each of these 200 rendered images, we computed the mean CIE xy image chromaticity and selected one whose mean chromaticity ([0.324, 0.359]) closely matched that of the image of the real scene under the target illumination.

To produce a stereo pair of images, the scene was rendered from two viewpoints, horizontally displaced by 6.4 cm; one corresponded to the left eye position and the other corresponded to the right eye position. The camera positions used for rendering approximated the observer's viewpoint in the real scene condition, and the stimulus box was seen by the cameras through an aperture in the front wall of the modeled stimulus box. The area visible to the cameras outside of the aperture was specified to have a black (nonreflective) surface reflectance function. Only the back wall and part of the floor of the stimulus box were visible from the camera viewpoints.

We rendered stereo pairs for 201 versions of the scene, with the different versions varying in terms of the spectral power distributions specified for the area lights. These illuminations matched the nominal illuminations used in the real scene condition. The target scene was rendered under the target illumination (Figure 2B) and 200 comparison scenes were rendered under each of the comparison illuminations (50 comparison illuminations × 4 chromatic directions of the illumination change; see Figure 3).

The stimulus scenes were rendered using Mitsuba renderer (https://www.mitsuba-renderer.org/), an open-source package that uses ray-tracing techniques to provide physically accurate image synthesis. Rendering was done via a path-tracer integrator (which enables realistic interreflections), a low discrepancy sampler (sample count 320), and using the RenderToolbox3 routines (Heasly, Cottaris, Lichtman, Xiao, & Brainard, 2014; https://github.com/DavidBrainard/RenderToolbox3/wiki), which facilitated the rendering and enabled us to specify the reflectance of each surface and the spectral power distribution of the illumination in each scene.

Each rendered stimulus scene was a 31-plane hyperspectral image. These hyperspectral images were converted into a three-plane LMS images by computing pixel-by-pixel excitations that would be produced in the human L-, M-, and S-cones using Stockman–Sharpe two-degree cone fundamentals (Stockman & Sharpe, 2000; CIE, 2007). We used monitor calibration data and standard colorimetric methods to convert these LMS images to RGB images for presentation (Brainard, Pelli, & Robson, 2002; Brainard & Stockman, 2010). Monitor calibrations included the measurements of spectral power distribution of the monitor's primaries and the gamma function of each monitor channel, made using a PR-670 SpectraScan radiometer (PhotoResearch).

The rendered images were scaled by a constant to maximize the used fraction of the display gamut. The effect of this scaling is equivalent to increasing the illumination irradiance by a common factor and preserves the relative equivalence of illumination irradiance across scenes. From the optical distance of 76.4 cm, the size of each stimulus image was 15.5° × 14.1° of visual angle (largest surface patch on the back wall was approximately 2.5° × 3.7°). Mean image luminance was 16.41 cd/m^2^ (all xyY values we report for simulated image are computed by averaging values from the left and right images).

**Figure A1:**
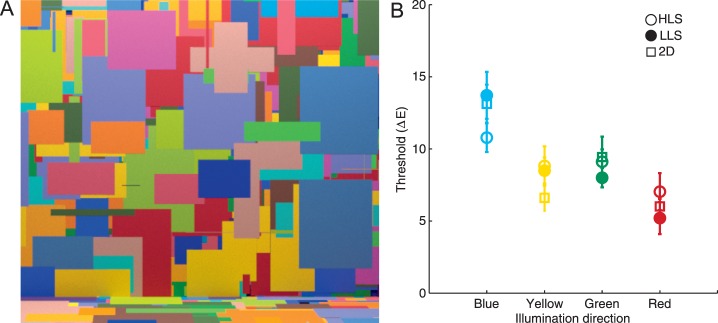
Experiment A. (A). Stimulus scene rendered under target illumination (left eye position). (B) Mean discrimination thresholds for four directions of illumination change in the HLS (open circles), LLS (filled circles), and 2D condition (open squares). Error bars represent ±1 *SEM*.

To produce Figures 2, 3, and A1, which are used here for illustration purposes, the hyper-spectral stimulus images were converted to CIE xyY, tone-mapped, and then converted to sRGB (International Electrotechnical Commission [IEC], 1999). The tone mapping was achieved by choosing an arbitrary maximal luminance (four times the mean luminance of one of the images) and truncating (in all images) the luminance of all pixels that were higher in luminance than the maximal value. In performing the tone mapping, pixel chromaticity was preserved. The images were then converted to a linear sRGB primary representation and scaled by a common factor so that they used the full gamut of the sRGB space. The scaled images were gamma corrected for display according to the sRGB standard. Tone mapping was not applied to the experimental stimuli.

### Experimental procedures

The experimental procedures were similar in the real scene and simulated scene conditions.

In the real scene condition, the observer was asked to sit as close as possible to the lightroom porthole and look into the stimulus box. The observer's head was not fixed. Before the start of the experiment, observers were dark-adapted for 2 min. Each trial of the experiment consisted of three intervals: the stimulus box was first presented under the target illumination (2000 ms). This interval was followed by two successive presentations of the stimulus box. One of these presentations was under a comparison illumination, while the other was under the target illumination (500 ms each, order chosen randomly on each trial). The observer's task was to indicate which of these two presentations had an illumination most similar to the target. Each presentation was preceded by a beep and followed by a 400-ms dark interval. After the stimulus presentations, the dark interval continued until the observer responded by pressing a button on a game controller. A new trial then started immediately. Observers were given a break after every 100 trials, followed by another dark adaptation period of 2 min.

Within each block of trials, 12 interleaved 1-up–2-down staircases on the comparison illumination were run (3 independent staircases for each of the 4 chromatic directions of illumination change). The value of the comparison illumination on the first trial of a staircase was chosen randomly from a predetermined interval that differed across the three staircases (11–20, 21–30 or 31–40 Δ*E* units). The staircase step size at the beginning of the trial was set to 15 and changed after each of the first four reversals (to 10, 5, 3, and finally to 1 Δ*E* nominal unit). The staircase terminated after the sixth reversal, or after 50 trials if a sixth reversal was not reached. Within a block, staircases were interleaved and presented in random order, until all had been completed. The stimulus range available to the staircases included 0 Δ*E* units, where there was no difference between the target and comparison illuminations. A block of trials typically lasted 30–40 min.

The procedure for the simulated scene condition was closely matched to that for the real scene condition. On each trial, the observer first saw the target scene, followed by two subsequent scenes: one was a repeat of the target scene and one was a scene illuminated by a comparison illumination. The observer's task was to indicate which of the two subsequent scenes was illuminated most similarly to the initially presented target scene. Observers typically completed a block of trials in about 30 min.

There were a few small procedural differences between the real and the simulated scene conditions: (a) The exact wording of instructions differed slightly (instructions verbatim for both conditions are available in the online supplement). Further, in the simulated scene condition (b) the minimum value used in the staircases was 1 rather than 0 nominal Δ*E* units; (c) each stimulus presentation interval was 170 ms longer than its real condition counterpart; (d) observers were not dark-adapted before the experiment; (e) observers were not given formal breaks within a block of trials; instead, they were encouraged to take breaks as needed (to take a break, the observer was instructed to remember the response he or she would give, but delay entering it until ready to continue); (f) in the real scene condition, each observer completed one block of trials; in the simulated scene condition, each observer completed two blocks of trials, each on a different day; and (g) in the real scene condition, the observers completed an additional block of trials in which objects were placed in the stimulus box (three matte plastic spheres, suspended by black rods); these data are not reported here.

#### Observers

Twelve observers participated in the real scene condition (eight men, four women; aged 19–25). Ten observers (five men, five women; aged 19–21) participated in the simulated scene condition. All observers had normal or corrected-to-normal visual acuity according to observer self-report (Newcastle) or as assessed via a Snellen chart (University of Pennsylvania, 20/40 or better in both eyes) and normal color vision (both Newcastle and Penn, 0 plates incorrect on Ishihara color plates; Ishihara, 1977). Observers in the simulated scene condition were also required to have normal depth perception from stereopsis, assessed via a custom procedure (see Lee & Brainard, 2014, for details).

The observers were recruited at Newcastle University (real scene condition) or the University of Pennsylvania (simulated scene condition). They received course credit or payment for their participation. All experimental procedures were approved by the Newcastle University Ethics Board (real scene condition) or the University of Pennsylvania Institutional Review Board (simulated scene condition), and were in accordance with the World Medical Association Declaration of Helsinki.

#### Data analysis

We examined different ways of analyzing the staircase data. For example, one could first find a threshold for each staircase by averaging the stimulus values at reversals at the lowest step size and then calculate the mean thresholds across the three staircases run for each illumination change direction. Preliminary analysis of the data revealed, however, that some staircases fail to converge. Thus, instead of averaging reversals, we found a discrimination threshold for each illumination change direction, for each block and observer, as follows. We aggregated all trials from all three staircases for the chromatic direction, ordered them by illumination change value (Δ*E*) and grouped them into bins of 10 trials each. If the total number of trials was not divisible by 10, the last bin contained the remaining trials. For each bin, we computed the average size of the illumination change value as well as the proportion of correct trials. We then fitted a psychometric function (cumulative Weibull) to the binned data and extracted a threshold by finding the illumination change value that corresponded to 70.71% correct identification (a recommended threshold value for the 1-up–2-down staircase procedure; Wetherill & Levitt, 1965). To fit the Weibull function, we used routines provided in the Palamedes Toolbox (Version 1.8; Prins & Kingdom, 2009, http://www.palamedestoolbox.org/). The guess rate was fixed at 0.5, corresponding to chance performance in our task, while the lapse rate was allowed to vary between 0 and 0.05.

In the simulated scene condition in which observers ran two blocks of trials, the threshold value for each illumination direction was obtained by averaging thresholds from the two blocks. For some observers in the simulated scene conditions, the threshold obtained for at least one of the blocks and at least one illumination change direction fell outside of the stimulus range (<1 or >50). Data for these observers was excluded from further analysis. There was one such observer in Experiment 1 (male, age 21) and two in Experiment A1 (condition LLS; see below), out of 58 observers in total who participated in our experiments.

#### Online supplement

For all experiments, the online supplement (http://color.psych.upenn.edu/supplements/illuminationdiscrimination1/) provides instructions verbatim, tables specifying the difference between the target and comparison illuminations in CIELUV Δ*E*, stimulus specification, and individual observer data. Stimulus information includes spectral power distributions of all experimental illuminations and the light emitted by each LED channel (real scene condition), a Blender file specifying stimulus geometry, surface reflectance functions, and illumination spectra, as well as RenderToolbox3 mapping files used for rendering (simulated scene conditions).

### Results

**Figure 4:**
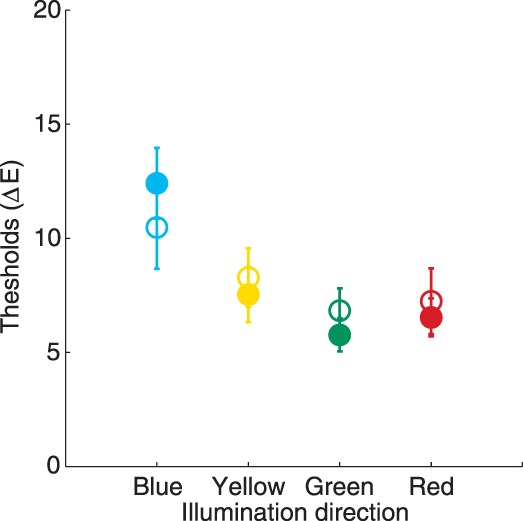
Experiment 1: Results. Mean discrimination thresholds for four directions of the illumination change in the real scene condition (filled circles) and simulated scene condition (open circles). Error bars represent ±1 *SEM*.

Figure 4 shows mean discrimination thresholds across the four chromatic directions of illumination change (averaged across observers) for the real scene (filled circles) and for the simulated scene (open circles). The sensitivity to changes in illumination across different directions was essentially identical in the two conditions. A two-way analysis of variance (ANOVA) with scene condition (real vs. simulated) as a between-observers factor and illumination direction (4 levels) as a within-observer factor did not reveal a significant main effect of condition or Condition × Illumination Direction interaction, *F*(1, 19) = 0.01, *p* = 0.9 and *F*(2, 37.3) = 0.96, *p* = 0.4, respectively. The sensitivity to illumination changes, however, varied across different chromatic directions: main effect of illumination, *F*(2, 37.3) = 10.41, *p* < 0.001.

To further investigate differences in thresholds measured for different directions of illumination change we separately conducted paired *t* tests. To correct for multiple comparisons, we adjusted significance levels using Bonferroni correction: for six pairwise comparisons the adjusted alpha level for a corrected *p*-value of 0.05 is 0.0083 (0.05/6). Consistent with the results of Pearce et al. (2014), we find that that sensitivity was the worst for blue illumination changes: thresholds for blue illumination were the highest and significantly higher than for green and for red directions, *t*(20) = 4.36, *p* < 0.001 and *t*(20) = 4.79, *p* < 0.001, respectively. Differences in thresholds across other chromatic directions were not significant.

In Appendix C we repeat the analyses we report here using a larger sample size in the simulated scene condition (*N* = 19), combined across experiments. This extended analysis leads to the same conclusions as those we report here.

## Experiment 2

In Experiment 2, we investigated whether sensitivity to changes in illumination across different chromatic directions depends on the ensemble of surfaces in the scene. We measured illumination discrimination thresholds for three different simulated scenes. Each scene had the same spatial structure, but the reflectances assigned to the individual surfaces differed, resulting in different average scene chromaticities. One of the scenes we used was identical to that from Experiment 1, and we continue to label it as the neutral scene. Based on the appearance of the other two scenes under the target illumination, they were labeled as the reddish-blue and yellowish-green scenes. Figure 2 shows the three scenes rendered under the target illumination.

### Methods

#### Stimuli

The geometry of the stimulus images was the same across the three conditions. The distribution of surface reflectances, however, differed across the scenes. One scene was the neutral scene (mean CIE xy chromaticity [0.323, 0.357]), essentially the same as that of the simulated scene used in Experiment 1 (Figure 2B). We then selected two other sample scenes from the 200 candidate scenes that were produced as part of choosing the Experiment 1 simulated scene. These had different chromaticities: [0.361, 0.339] (reddish-blue scene, Figure 2C) and [0.399, 0.425] (yellowish-green scene, Figure 2D). For each of the three scenes (neutral, reddish-blue, and yellowish-green) we then rendered a full stimulus set (201 pairs of images) and measured illumination discrimination thresholds for each scene condition.

As in Experiment 1, in preparation of stimuli for presentation all images were scaled by the same factor to maximize the portion of the display gamut used. The mean image luminance differed slightly across scene conditions because of the difference in surface reflectances across the three scenes. Mean luminances of the target scene for the neutral, reddish-blue, and yellowish-green scene were, respectively, 16.51, 15.29, and 24.34 cd/m^2^. Due to an error in the experimental code, the size of the presented stimulus images was larger than in Experiment 1 (18.56° × 17.27°), which in turn reduced the overall depth in the stimuli. A control condition (see Appendix B) shows that this difference in depth did not affect measured illumination discrimination thresholds.

#### Observers

Ten observers participated in the experiment (three men, seven women; ages 18–21). Observers completed six blocks of trials in total (two per scene condition). The order in which blocks for different scene conditions were run was counterbalanced across observers. The observers completed one block of trials for each scene condition first, with the second set of blocks run in the same order as the first. Two observers also completed an additional (third) block of trials in the neutral scene condition near the end of the second block.

### Results

**Figure 5:**
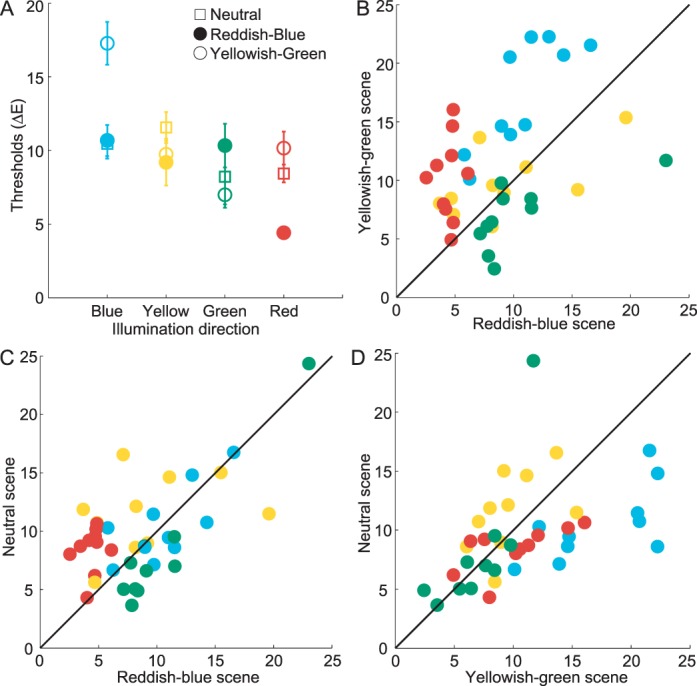
Experiment 2: Results. (A) Mean discrimination thresholds for four directions of illumination change (across observers) are shown for three scene chromaticities (neutral = squares, reddish-blue = filled circles, yellowish-green = open circles). Error bars represent ±1 *SEM*. (B, C) Comparison of illumination discrimination thresholds for different scene chromaticities for individual observers: yellowish-green versus reddish-blue (B), neutral versus reddish-blue (C), and neutral versus yellowish-green (D). Diagonal indicates the identity line. Colors of the symbols (blue, yellow, green, red) correspond to illumination change directions.

For each observer, we computed discrimination thresholds for each illumination change direction in each trial block following the same data analysis methods we used in Experiment 1. We then found the mean thresholds for each scene type and illumination direction by averaging thresholds across blocks. Mean illumination discrimination thresholds (averaged across observers) are shown in Figure 5A.

As in Experiment 1, we found that discrimination thresholds varied across different chromatic directions of illumination change, as shown by a two-way repeated measure ANOVA with scene chromaticity and illumination direction as within-observer factors (main effect of illumination, *F*(1.7, 15.1) = 8.43, *p* < 0.01). Importantly, the sensitivity to illumination changes across different directions depended on scene chromaticity (main effect of scene chromaticity, *F*(1.3, 11.8) = 13.50, *p* < 0.01; Scene Chromaticity × Illumination Direction interaction, *F*(6, 54) = 12.50, *p* < 0.001).

To further explore how illumination discrimination changes with the variation in scene chromaticity, we compared the thresholds for each illumination direction across the three scene types using separate paired *t* tests. To correct for multiple comparisons, we used Bonferroni correction, adjusting the significance level (for a corrected *p*-value of 0.05) to 0.0042 (0.05/12). The variation in scene chromaticity implemented in our experiment had a strong effect on blue and red discrimination thresholds. In the red direction, thresholds were the lowest for the reddish-blue scene and significantly lower than those in the neutral or yellowish-green scenes: *t*(9) = 6.56, *p* < 0.001; *t*(9) = 6.33, *p* < 0.001, respectively. In the blue direction, thresholds were the highest in the yellowish-green scene and significantly higher than for the neutral or the reddish-blue scene: *t*(9) = 6.33, *p* < 0.001; *t*(9) = 7.65, *p* < 0.001, respectively. We also noted a trend for the green illumination direction: here, thresholds for the reddish-blue scene were higher than those for the neutral or the yellowish-green scene. For our sample, however, this trend did not reach the adjusted significance level: *t*(9) = 3.58, *p* = 0.006; *t*(9) = 3.10, *p* = 0.013, respectively.

Analysis of individual observer data further confirms the results from the post hoc tests. Panels B–D of Figure 5 compare thresholds across our three scenes for each observer and show that the effects we report for the red and blue illumination change held for all observers. The trends we noted for the green illumination change held for all but two observers (one per comparison).

## Discussion

We measured sensitivity to changes in illumination along four different chromatic directions (blue, yellow, red, and green) in two different types of stimulus scenes: the real illuminated scene and a simulated scene that closely matched the real scene. The real and simulated scenes had similar layout, used overlapping sets of surface reflectances and had similar average chromaticity. Illumination discrimination thresholds were essentially identical across the two scenes, indicating that simulated scenes provide a valid laboratory model for understanding the type of illumination discrimination studied here. This finding provides a foundation for future studies that can take full advantage of simulated stimulus manipulations to probe processes that underlie illumination discrimination. Our study replicated the main findings reported by Pearce et al. (2014), which employed a real scene setup that was nearly identical to ours. First, sensitivity to changes in illumination, as assessed using the CIELUV Δ*E* metric, varied across different chromatic directions. Second, for a scene that had roughly neutral chromaticity, sensitivity was the worst for blue illumination changes and significantly lower than for either red or green illumination changes. These results held in both the main and extended variant of our analysis (Appendix C), in which the latter includes a larger sample of observers (combined across Experiments 1 and 2). One difference between our study and that of Pearce et al. (2014) is that we find better discrimination for the red illumination change direction than for yellow, while they found the opposite. This difference was not significant in either the Pearce et al. (2014) study or in our main analysis, but it did reach significance in the extended analysis. Similarly, while Pearce et al. (2014) found significantly better discrimination for yellow than blue illumination changes, this difference did not reach significance in our study (in either the main or the extended analysis). Given the differences in design and analysis between the two studies (e.g., Pearce et al. [2014] tested differences in discrimination accuracy rather than differences in thresholds), we view the overall pattern of results as satisfyingly consistent across the two studies. It is not surprising that differences in experimental power lead to small variations in which pairwise comparisons rise to significance.

Although our real and simulated scenes were well matched in chromaticity, they differed in luminance: based on estimates from the hyperspectral images of the real scene, the mean luminance of the simulated scene was about twice as high as that of the real scene. In a control experiment (Appendix A), we show that illumination discrimination thresholds (either relative or absolute) do not vary with mean luminance over the range we studied.

Using simulated scenes, we then investigated whether the relative sensitivity to different directions of illumination change depends on the surfaces in the scene. The following example illustrates why such effects might be expected. Consider a hypothetical “red” illumination change that consists of an increase in illumination power solely at wavelengths greater than 600 nm. If there are no surfaces in the scene that reflect light at these wavelengths, then the hypothetical illumination change will not result in a change in the retinal image, and will therefore go undetected. Thus, changing from our neutral surface ensemble, where all surfaces reflect light at some wavelengths greater than 600 nm to a hypothetical ensemble in which no surface reflects wavelengths greater than 600 nm, would result in a large decrease in sensitivity to the hypothetical “red” illumination change. Although our actual changes are not as extreme, the general patterns we observe are consistent with the intuition provided by this example.

We show that varying the surface scene ensemble in a manner that changes the average chromaticity of the scene modulates the discriminability of changes in illumination across different chromatic directions. Specifically, as the number of surfaces that appear red and blue increases in the reddish-blue scene, observers' sensitivity to changes in the red illumination direction increased relative to that measured for the neutral and yellowish-green scenes. Similarly, as the number of surfaces that appear green and yellow increased in the yellowish-green scene, illumination discrimination thresholds for the blue illumination change directions increased relative to those measured for the neutral and reddish-blue scene. These shifts in thresholds were observed for all observers. A dependence of illumination discrimination thresholds on stimulus content has also been reported by Álvaro, Lillo, Moreira, Linhares, and Nascimento (2015). They measured illumination discrimination along the blue and yellow chromatic directions in natural scenes in red–green dichromats and normal observers and found that thresholds differed significantly across scenes.

Previous studies have also reported that illumination discrimination depends on characteristics of the stimulus scene. For example, Zaidi, Spehar, and DeBonet (1997) studied illumination discrimination in simple two-dimensional scenes and showed that sensitivity is significantly better for spatially uniform than for variegated patterns. Similarly, Pearce et al. (2014) found better illumination discrimination in real three-dimensional scenes when the room walls were lined with homogenous gray rather than a colorful Mondrian wallpaper, but that introducing different objects (either novel or familiar) into the Mondrian-lined scene did not significantly affect discrimination. Our results extend these findings and contribute to our understanding of stimulus factors that affect illumination discrimination in complex scenes. The clear relationship between the set of surfaces in the scene and illumination discrimination suggests that any characterization of sensitivity to changes in illumination must be defined relative to the set of surfaces in the scene in which it was measured.

We show that, overall, observers are able to discriminate fine chromatic changes in illumination in the stimulus scenes we used. It remains an open question, though, which mechanisms the human visual system uses to extract and process information about the changes in illumination. One possibility is that observers' performance is based on a global surface-independent estimate of the illumination. Alternatively, observers might compare overall image chromaticities independently of illumination representations, or track and compare the images of an individual surface or subset of surfaces. Further research is required to distinguish between these (and perhaps other) possibilities.

**Figure D1:**
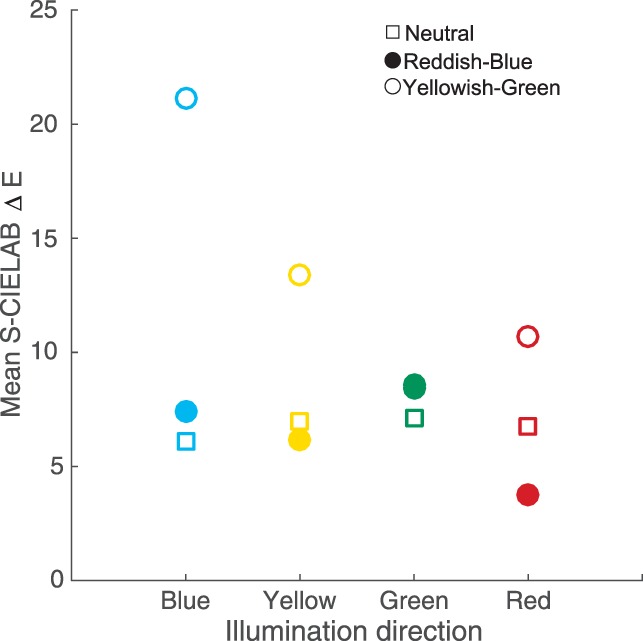
Image differences at threshold in S-CIELAB ΔE (Experiment 2). For each scene type and illumination change direction we show the size of the difference (expressed as a mean S-CIELAB Δ*E*) between the target image and the comparison image closest to threshold measured in Experiment 2. Colors of the symbols correspond to illumination change directions; the neutral scene is shown in square symbols, reddish-blue in filled circles, and yellowish-green in open circles.

We did, however, ask the simple question of whether an established color image difference metric that takes image spatial structure into account (S-CIELAB; Zhang & Wandell, 1997) could account for our threshold data. We used the data from Experiment 2. If the S-CIELAB metric is a good predictor of observers' thresholds across different illumination change directions and different stimulus scenes, then the S-CIELAB image difference between target and comparison images at threshold will be roughly the same for all scenes and illumination-directions. As we show in Figure D1 (Appendix D) this is not the case: the size of the mean S-CIELAB image differences at threshold varies over a fairly large range. We conclude that our illumination discrimination thresholds are not predicted by the known features of human spatio-chromatic image discrimination embodied in the S-CIELAB metric.

Characterizing humans' ability to perceive spatial and temporal changes in illumination provides information about the visual system's representation of properties of the distal stimulus. Despite its importance and its implications for understanding how vision extracts information about object properties such as shape, color, or material, illumination perception has not been a major focus of study. This trend is now changing and, in recent years, how the visual system represents illumination has become an active area of investigation. For example, one line of research has focused on developing models of perceived illumination from indirect measurements, based on tasks in which the observers judge object shape (Morgenstern, Murray, & Geisler, 2010; van Doorn, Koenderink, Todd, & Wagemans, 2012) or reflectance (Boyaci, Maloney, & Hersh, 2003; Fleming, Dror, & Adelson, 2003; Bloj et al., 2004; Boyaci, Doerschner, & Maloney, 2006; Logvinenko & Maloney, 2006). Another line directly probes different aspects of illumination perception, such as sensitivity to direction of illumination (Pont & Koenderink, 2007; Morgenstern, Geisler, & Murray, 2014), perception of spatial distribution of illumination in complex scenes (Xia, Pont, & Heynderickx, 2014; Kartashova, Sekulovski, de Ridder, te Pas, & Pont, 2016), or dependence of perceived illumination intensity on the ensemble of surfaces in a scene (Rutherford & Brainard, 2002). The relationship between explicit perception of illumination and perception of intrinsic object properties remains an interesting open question (for reviews, see Brainard & Maloney, 2011; Murray, 2013; Fleming, 2014).

Our results show that for well-matched stimulus scenes, observers' performance in an illumination discrimination task is essentially identical for real and simulated scenes. We interpret this as a finding specific to our stimuli and task, rather than a general statement about the correspondence of color appearance measurements obtained with real and simulated scenes. The question of whether and to what extent simulated scenes capture visual experience of the real scenes is longstanding (Koenderink, 1999), particularly in the domain of color and lightness (Hurlbert, 1998a). Consistent with our current findings, some studies have shown that for well-matched real and simulated scenes with little geometric structure in the illumination, there is good agreement between results obtained using the two types of stimuli (e.g., Agostini & Bruno, 1996; McNamara, Chalmers, Troscianko, & Gilchrist, 2000). Other studies suggest that for different classes of stimuli, which typically include multiple regions of illumination within a single scene, the results obtained using simulated scenes systematically deviate from those obtained with real illuminated objects (e.g., Radonjić, Todorović, & Gilchrist, 2010; Lee & Brainard, 2014). It remains a crucial question for future research to characterize the conditions under which results obtained with simulated scenes accurately predict performance for the real scenes they model. What we can conclude from our current work is that for the scenes like those we studied, graphics simulations support performance which would be found for similar real world scenes.

## Supplementary Material



## Figures and Tables

**Figure B1:**
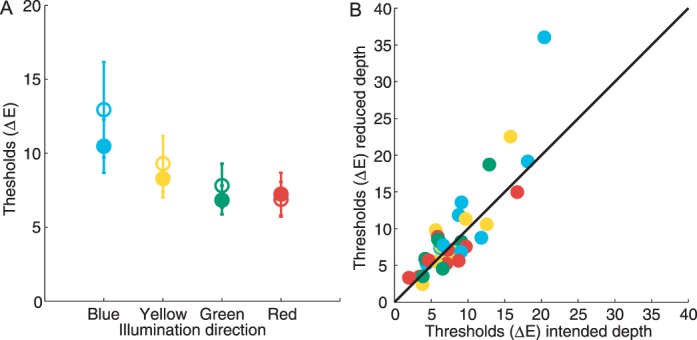
Experiment B: Results. (A) Mean discrimination thresholds for four directions of illumination change in the intended (filled circles) and reduced depth conditions (open circles). Error bars represent ±1 *SEM*. (B) For each observer, illumination discrimination thresholds measured in the reduced depth condition are plotted against those measured in the intended depth condition. Colors of the symbols correspond to illumination change directions.

## Appendix A: Illumination discrimination thresholds do not depend on stereoscopic presentation or the overall stimulus luminance level

We ran a control experiment to test whether the measured illumination discrimination thresholds depend on either the perceived depth in the stimulus scene or the overall stimulus luminance level. This experiment consisted of three presentation conditions, each run with a different group of observers. All three conditions used the same stimuli (Figure A1A), but the stimulus presentation differed. In the high-luminance stereoscopic condition (HLS), the presentation conditions were identical to those in Experiments 1 and 2. The observers viewed the scene stereoscopically and the stimulus images were scaled to use most of the monitor's display gamut (mean target scene luminance: 24.36 cd/m^2^). To test whether thresholds are sensitive to overall scene luminance, in the low-luminance stereoscopic condition (LLS), the stimuli were scaled down in overall luminance (mean target scene luminance: 4.82 cd/m^2^). To test for the effect of stereoscopic presentation, we also replicated the experiment using a single screen and the high luminance stimuli (2D condition). The 2D stimulus set only included the left images from the stereo pairs. These were scaled for presentation to fit the gamut of a different display, as described below, and had even higher luminance than the stimuli in HLS condition (mean target scene luminance: 35.90 cd/m^2^).

The methods were similar to those used in Experiments 1 and 2, with the differences noted below.

### Apparatus

The apparatus used to display the stimuli in the HLS and LLS condition was the same as that used in Experiments 1 and 2. In the 2D condition, the stimuli were displayed on a 23 in. monitor (ASUS Proset LCD PA 238) monitor, driven at a pixel resolution of 1920 × 1080, a refresh rate of 60 Hz, and with a 10-bit resolution for each RGB channel via a PNY 600 graphics card. The monitor was fitted with a black hood that extended out by approximately 20 cm from the top and sides of the screen. Observers viewed the stimuli from approximately 45 cm distance. The observer's head was fixed using a chin rest with height adjusted for comfort. The size of the stimulus image on the screen was 14.2 cm height × 12 cm width (17.9° × 15.2° of visual angle; the largest surface on the back wall approximately 3° × 4°).

### Stimulus

The geometry of the stimulus scene was the same as that used in the Experiments 1 and 2. The surface reflectance functions assigned to surfaces in the scene were different and included 18 chromatic surface reflectance samples from the Macbeth color checker chart (obtained at http://www.babelcolor.com and provided as part of the RenderToolbox3 distribution; Figure A1A). The file specifying the mapping of surface reflectances to each surface in the stimulus scene is available in the online supplement. The mean xy chromaticity of the stimulus image under the target illumination was [0.36, 0.35].

For the 2D condition of the experiment, the rendered hyperspectral images were converted into three-plane CIE XYZ images via the CIE XYZ 1931 color matching functions (CIE, 1932). These were then converted into RGB images for presentation using monitor calibration data (calibration performed using a Konica Minolta CS2000 spectroradiometer and standard colorimetric methods as described above). To fit the monitor gamut, the stimulus images were scaled in intensity and slightly modified (top 29 rows of pixels were cropped off to eliminate a Mondrian surface that fell outside of the monitor gamut). In addition, the range of comparison scenes that was used in the 2D condition was reduced to 30 per illumination direction (1–30 nominal Δ*E* steps).

### Observers

Twenty-six observers participated in the experiment: seven in the HLS condition (four men, three women; ages 18–21, University of Pennsylvania); 10 in the LLS condition (seven men, three women; ages 18–21, University of Pennsylvania); and nine in the 2D condition (four men, five women; ages 21–29, Newcastle University). Two out of the 10 observers from the LLS condition (both male, ages 19 and 20) were excluded from the analysis because their thresholds for at least one chromatic direction (in at least one session) fell outside of the stimulus range (see data analysis section above).

Observers in the 2D condition completed one block of trials, while the observers in the HLS and LLS conditions completed two blocks of trials each. Similar to the real scene condition of Experiment 1, the observers who participated in the LLS condition also completed two additional blocks of trials with a slightly different stimulus scene, which included objects (five spheres floating in midair). These data are not reported here.

### Results

The mean illumination discrimination thresholds for each condition are shown in Figure A1B (HLS = open circles, LLS = filled circles, 2D = open squares). The thresholds did not differ across the presentation conditions. A two-way ANOVA with presentation condition (LLS, HLS, 2D) as a between-observers factor and chromatic direction of illumination change as a within-observer factor did not show a main effect of condition on illumination discrimination thresholds or a Condition × Illumination Direction interaction: *F*(2, 21) = 0.01, *p* = 0.99 and *F*(6, 63) = 1.98, *p* = 0.08, respectively. Consistent with the results from the previous experiments, the main effect of illumination was significant, *F*(3, 63) = 25.63, *p* < 0.001.

We further investigated differences in thresholds measured for different illumination change directions via *t* tests, corrected for multiple comparisons (using Bonferroni correction, see Experiment 1). We found that sensitivity was the worst for blue and best for red illumination changes. Thresholds for the blue illumination change direction were significantly higher than for green, red or yellow directions, *t*(23) = 5.10, *p* < 0.001; *t*(23) = 8.03, *p* < 0.001, *t*(23) = 5.07, *p* < 0.001, respectively, while thresholds for the red direction were significantly lower than for any other direction: red versus green: *t*(23) = 3.89, *p* = 0.001; red versus yellow: *t*(23) = 2.98, *p* = 0.007.

The finding that illumination discrimination is the poorest for the blue illumination change direction is generally consistent with the results of Pearce et al. (2014), as well as our Experiment 1, while the result that thresholds for the red illumination change are lowest differs. However, in comparing the results across experiments it is important to keep in mind that ensemble of surface reflectances in Experiment A1 differed from that used in Experiment 1 (and by Pearce et al., 2014). As we show in Experiment 2, the relative sensitivity across different illumination change directions depends strongly on surface scene ensemble; thus, a difference in the pattern of thresholds across illumination change directions between Experiments 1 and A1 is not surprising.

## Appendix B: Illumination discrimination thresholds across different stereoscopic depths

Due to an error in the experimental code, in some of our experiments that used simulated scenes (Experiment 2 and stereoscopic conditions of Experiment A1) the size of the displayed stimuli were larger than intended and inconsistent with the size specified in the 3D scene model. This caused the depicted stereoscopic depth relations in the scene to deviate from those specified in the model, with the effect being a reduction of depth. Specifically, the intended distance of the back wall from the eyes/cameras was 76.4 cm, but this was reduced to 50.8 cm and the intended depth of the stimulus box (front window to back wall) was 34.6 cm, but this was reduced to 20.5 cm. We therefore ran a control condition to test whether this difference in depicted stereoscopic depth had an effect on illumination discrimination thresholds. Specifically, in parallel with the simulated scene condition of Experiment 1, in which depth relations were displayed as intended, the observers also completed the illumination discrimination task for a set of stimuli in which overall depth was reduced.

Methods were identical to those used in the simulated scene condition of Experiment 1 and used the same 10 observers (out of which one was excluded from the analysis as noted above). Each observer completed four blocks of trials in total: two in which the image size was as intended (15.5° × 14.1° of visual angle; data shown in Experiment 1) and two in which the displayed image size was larger (19.4° × 17.6°; the reduced depth condition). Each block of trials was run on a different day, with an alternating order of conditions: half of the observers started with the intended depth condition, while the other half started with the reduced depth condition. The data from the first session was not saved for the first five observers we ran due to a bug in the experimental code. These observers continued the experiment as planned but ran an extra session at the end to make up for the lost data.

Figure B1A shows mean discrimination thresholds for the four directions of illumination change (averaged across observers) in the two depth conditions. Consistent with the findings of the experiment in Appendix A, which showed that illumination discrimination thresholds do not change in the absence of stereoscopic presentation, we find that thresholds in the two conditions were essentially identical. A two-way repeated measure ANOVA with the depth condition and illumination direction as within-observer factors did not reveal a main effect of condition or a significant Condition × Illumination direction interaction: *F*(1, 8) = 1.45, *p* = 0.26; *F*(1.4, 11.2) = 1.66, *p* = 0.2, respectively. As in all previous experiments we report, the main effect of the illumination direction was significant, *F*(3, 24) = 5.91, *p* < 0.01.

Comparing illumination discrimination thresholds for individual observers confirms the results of the group analysis. In Figure B1B the illumination discrimination thresholds measured in the intended depth condition are shown against those from the reduced depth condition. With the exception of one outlying point, thresholds for all observers and illumination directions group close to the identity line.

## Appendix C: Illumination discrimination in real and simulated scenes: extended data set

The observers in the simulated scene condition of Experiment 1 viewed the same neutral stimulus scene as the observers in Experiment 2. Although the images presented in Experiment 2 were slightly different in size (resulting in reduced scene depth), the results of the control condition described in Appendix B showed that this reduction in depth had no effect on illumination discrimination thresholds. We therefore combined observers' data for the neutral scene condition across the two experiments and replicated, with more statistical power, the analyses from Experiment 1.

We first averaged the data from the two depth conditions (Appendix B) for each of the nine (valid) observers from Experiment 1. We than added the data from the 10 observers from Experiment 2 (neutral scene condition) to the set and performed the same analyses as described in Experiment 1 (now with 19 observers in the simulated scene condition). The results were essentially identical as those we reported above:

- We found no difference in illumination discrimination in real and simulated scenes: main effect of condition, *F*(1, 29) = 1.04, *p* = 0.31; Condition × Illumination Direction interaction, *F*(2.6, 75.8) = 1.88, *p* = 0.12.
- Sensitivity to changes in illumination differed across different chromatic directions: main effect of illumination direction, *F*(2.6, 75.8) = 13.11, *p* < 0.001.
- The relative order of thresholds across illumination directions was the same across conditions and highest for the blue illumination change (then yellow, then red, then green).
- Thresholds for the blue illumination changes were significantly higher than for either the green or red illumination changes: *t*(30) = 4.18, *p* < 0.001; *t*(30) = 5.48, *p* < 0.001, respectively.

Unlike in Experiment 1, this analysis also revealed that thresholds for the yellow illumination change were significantly higher than those for both red and green illumination changes: *t*(30) = 2.87, *p* = 0.008 and *t*(30) = 2.9, *p* = 0.007, respectively.

## Appendix D: Can the S-CIELAB image metric predict illumination discrimination thresholds?

We used the stimulus set from Experiment 2 to investigate whether measured illumination discrimination thresholds can be predicted from the S-CIELAB image difference metric—an extension of CIELAB that includes a spatial filtering component to capture the spatio-chromatic contrast sensitivity of human vision (Zhang & Wandell, 1997). For each of the three stimulus scenes, we computed the difference in S-CIELAB Δ*E* units between the scene illuminated by target illumination (the target image) and a threshold image for each illumination change direction as follows. For each scene and illumination direction we first determined the threshold image by finding the comparison image in our stimulus set for which the difference between the simulated illumination used to render it and the target illumination is nearest to the average threshold value we measured. We then computed the mean S-CIELAB difference between the target and threshold image. Differences are computed pixel-by-pixel and we took the image difference to be the average of the pixel-by-pixel values taken across the whole image. If the S-CIELAB differences predict observers' thresholds accurately, then the image differences between the target and threshold images will be roughly the same for all scenes and illumination change directions. Figure D1, which shows the image differences at threshold, demonstrates that this is not the case: the S-CIELAB image differences vary widely across illumination change directions and scenes.
