# Supplementary figures and images for: Illumination discrimination in real and simulated scenes
Source: J Vis. 2016 Sep 1;16(11):2. doi: 10.1167/16.11.2 (PMC5024666; doi:10.1167/16.11.2)

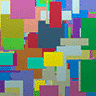

Supplement: Supplementary file 1 [file i1534-7362-16-11-2-icon01.gif]
